# Tissue-Specific Expressed Antibody Variable Gene Repertoires

**DOI:** 10.1371/journal.pone.0100839

**Published:** 2014-06-23

**Authors:** Bryan S. Briney, Jordan R. Willis, Jessica A. Finn, Brett A. McKinney, James E. Crowe

**Affiliations:** 1 Department of Pathology, Microbiology and Immunology, Vanderbilt University Medical Center, Nashville, Tennessee, United States of America; 2 Department of Pediatrics, Vanderbilt University Medical Center, Nashville, Tennessee, United States of America; 3 The Chemical and Physical Biology Program, Vanderbilt University, Nashville, Tennessee, United States of America; 4 The Vanderbilt Vaccine Center, Vanderbilt University Medical Center, Nashville, Tennessee, United States of America; 5 Tandy School of Computer Science and Department of Mathematics, University of Tulsa, Tulsa, Oklahoma, United States of America; King’s College London, United Kingdom

## Abstract

Recent developments in genetic technologies allow deep analysis of the sequence diversity of immune repertoires, but little work has been reported on the architecture of immune repertoires in mucosal tissues. Antibodies are the key to prevention of infections at the mucosal surface, but it is currently unclear whether the B cell repertoire at mucosal surfaces reflects the dominant antibodies found in the systemic compartment or whether mucosal tissues harbor unique repertoires. We examined the expressed antibody variable gene repertoires from 10 different human tissues using RNA samples derived from a large number of individuals. The results revealed that mucosal tissues such as stomach, intestine and lung possess unique antibody gene repertoires that differed substantially from those found in lymphoid tissues or peripheral blood. Mutation frequency analysis of mucosal tissue repertoires revealed that they were highly mutated, with little evidence for the presence of naïve B cells, in contrast to blood. Mucosal tissue repertoires possessed longer heavy chain complementarity determining region 3 loops than lymphoid tissue repertoires. We also noted a large increase in frequency of both insertions and deletions in the small intestine antibody repertoire. These data suggest that mucosal immune repertoires are distinct in many ways from the systemic compartment.

## Introduction

The humoral immune response produces a massively diverse repertoire of antibodies In order to respond effectively to challenge from a multitude of unfamiliar pathogens. Diversity in the primary (or, naïve) B cell repertoire is accomplished by combinatorial diversity that occurs following recombination of germline variable (V), diversity (D) and joining (J) germline genes and pairing of unique heavy and light chains [1]–[3]. Repertoire diversity is further enhanced in the memory repertoire by several affinity maturation processes including somatic hypermutation, which introduces point mutations and insertions/deletions (indels), and class-switching [4]–[6].

In studies of the circulating antibody repertoire, pathogenic infections have been shown to induce antibody responses with biased germline antibody variable gene use, and this bias is often maintained in the post-infection memory B cell population [7]–[12]. Since each individual has experienced a unique set of pathogenic encounters in a unique order, it is logical to expect that each individual might possess a uniquely biased memory repertoire that reflects the enrichment of clones specific for the particular history of pathogens. Surprisingly, however, circulating memory B cell repertoires often appear very similar when compared across individuals at the level of antibody variable gene usage, suggesting the presence of a global mechanism regulating the genetic composition of the peripheral blood antibody repertoire [13]–[16].

Circulating B cells with diverse surface receptors (that later become secreted antibodies with the same specificity) constitute the primary humoral immune cell type responding to systemic infection, and recent work has described the human peripheral blood antibody repertoire in great detail [13]–[15], [17], [18]. Much less is known about the repertoire composition of tissue-resident B cells, however. In the gut mucosa, resident plasma cells secrete almost exclusively IgA, and the presence of IgA-secreting plasma cells depends on the presence of colonizing bacteria in the gut [19]. In contrast to conventional germinal centers in lymph nodes and other lymphoid organs, many mucosal B cells are thought to mature using T cell-independent routes, which likely affects the diversity of the mucosal antibody repertoire [20], [21]. Indeed, spectratypic analyses of the mucosal antibody repertoire have provided evidence of increased oligoclonality of the mucosal IgA repertoire [22]. This finding raises the intriguing possibility that mucosal antibody repertoires are distinct from the peripheral blood repertoire, possibly because they are induced in response to site-specific pathogens or using unique maturation processes. Alternatively, there is substantial evidence that the B cell composition of the mucosa is different than peripheral blood, resulting in alterations in the expressed antibody repertoire. The presence of large numbers of commensals in the gut microbiome also could influence the specificity of the mucosal B cell repertoire. In this report, we used high-throughput DNA sequencing techniques to analyze the expressed antibody gene repertoire in order to determine whether mucosal lymphocytes harbor a unique repertoire. Indeed, a detailed analysis of mucosal and lymphoid repertoires revealed that mucosal antibody repertoires are genetically distinct from the antibody repertoires of both non-mucosal lymphoid tissues and peripheral blood cells.

## Materials and Methods

### Tissue-specific total RNA and mRNA

Purified polyA+ mRNA (lymph node) or total tissue RNA (all other samples) from the tissues of healthy human subjects was obtained from a commercial source (Clontech). Each RNA sample, as provided by Clontech, contains pooled RNA from multiple donors. The number of donors and demographic breakdown for each tissue donor pool is shown in Table 1.

**Table 1:** Demographics of pooled tissue sample donors from which the RNA pools were isolated.

Sample	# of donors	Age range (years)
Peripheral leukocytes	39	18–47
Bone marrow	56	22–85
Small intestine	15	29–61
Lung	13	32–61
Stomach	7	20–55
Lymph node	42	NA
Tonsil	34	22–61
Spleen	12	18–54
Thymus	25	NA

NA indicates age range was not available.

### cDNA synthesis and PCR amplification of antibody genes

100 ng of each mRNA or total RNA sample and 10 pmol of each RT-PCR primer (Table 2, adapted from [23]) were used in duplicate 50 µL RT-PCR reactions using the OneStep RT-PCR system (Qiagen). Thermal cycling was performed in a BioRad DNA Engine PTC-0200 thermal cycler using the following protocol: 50°C for 30 minutes, 95°C for 15 minutes, 35 cycles of (94°C for 45 seconds, 58°C for 45 seconds, 72°C for 2 minutes), 72°C for 10 minutes. cDNA synthesis and amplification was verified by agarose gel electrophoresis before duplicate RT-PCR reactions were pooled. 5 µL of each pooled RT-PCR reaction was used as template for 100 µL 454-adapter PCR reactions, which were carried out in quadruplicate. 20 pmol of 454-adapter primers (Table 2) and 0.25 units of AmpliTaq Gold polymerase (Applied Biosystems) were used for each reaction. Thermal cycling was performed in a BioRad DNA Engine PTC-0200 thermal cycler using the following protocol: 95°C for 10 minutes, 10 cycles of (95°C for 30 seconds, 58°C for 45 seconds, 72°C for 2 minutes), 72°C for 10 minutes.

**Table 2:** Primers used in RT-PCR and 454-Adapter PCR.

PRIMER NAME	SEQUENCE	PRIMER TYPE
V_H_1/7-FR1	*CCATCAG* CGTGTCTCTAGGCCTCAGTGAAGGTCTCCTGCAAG	RT-PCR
V_H_2-FR1	*CCATCAG* CGTGTCTCTAGTCTGGTCCTACGCTGGTGAACCC	RT-PCR
V_H_3-FR1	*CCATCAG* CGTGTCTCTACTGGGGGGTCCCTGAGACTCTCCTG	RT-PCR
V_H_4-FR1	*CCATCAG* CGTGTCTCTACTTCGGAGACCCTGTCCCTCACCTG	RT-PCR
V_H_5-FR1	*CCATCAG* CGTGTCTCTACGGGGAGTCTCTGAAGATCTCCTGT	RT-PCR
V_H_6-FR1	*CCATCAG* CGTGTCTCTATCGCAGACCCTCTCACTCACCTGTG	RT-PCR
J_H_ Consensus	*CGCTCAG* AGCACTGTAGCTTACCTGAGGAGACGGTGACC	RT-PCR
454-Adapter-A	CGTATCGCCTCCCTCGCG*CCATCAG* CGTGTCTCTA	454 PCR
454-Adapter-B	CTATGCGCCTTGCCAGCC*CGCTCAG* AGCACTGTAG	454 PCR

Multiplex identifiers (MIDs) are underlined. Forward primers use MID7 and reverse primers use MID4 (Roche). Additional complementarity between RT-PCR primers and 454-Adapter PCR primers is identified in italics. All primer sequences are shown in the 5′ to 3′ orientation.

### Amplicon purification and quantification

Amplicons were purified from the 454-adapter PCR reaction mix using the Agencourt AMPure XP system (Beckman Coulter Genomics) according to the manufacturer’s standard protocol. Purified amplicons were analyzed on a 2% agarose gel to verify complete primer removal and appropriate amplicon size before quantification using a Qubit fluorometer (Invitrogen, High-Sensitivity dsDNA kit) following the manufacturer’s standard protocol.

### 454 method sequence analysis of amplicons

The amplicon libraries were quantified using a Qubit fluorometer (Invitrogen, CA). The size and quality of the DNA libraries were evaluated on an Agilent Bioanalyzer 2100 using the DNA 7500 labchip (Agilent, Palo Alto, CA). The samples then were diluted to a working concentration of 1×10^6^ Molecules per µL. Quality control of the amplicon libraries and emulsion-based clonal amplification and sequencing on the 454 Genome Sequencer FLX Titanium system were performed by the W. M. Keck Center for Comparative and Functional Genomics at the University of Illinois at Urbana-Champaign according to the manufacturer’s instructions (454 Life Sciences, Branford, CT). Signal processing and base calling were performed using the bundled 454 Data Analysis Software version 2.5.3 for amplicons. Raw sequencing data will be available at the NCBI Sequence Read Archive (http://www.ncbi.nlm.nih.gov/sra) on the date of publication.

### Antibody Sequence Analysis

The FASTA files resulting from the 454 method sequence analysis were submitted to IMGT HIGH/V-Quest (IMGT, the international ImMunoGeneTics information system; www.imgt.org; founder and director: Marie-Paule LeFranc, Montpellier, France) [24], [25]. The sequences were analyzed using the HIGH/V-Quest option to perform codon-based correction of homopolymer-induced insertion/deletion errors, which are relatively common when using pyrosequencing. The IMGT output was parsed into a custom MySQL database for further analysis. Following initial analysis by IMGT, sequences were retained for analysis only if they passed additional antibody-related filters: (1) appropriate read length (>300 bases), (2) identification of V, D, and J segments by IMGT analysis, and (3) presence of an in-frame junctional rearrangement. To reduce the effect of high copy-number plasma cells on the repertoire analysis, identical sequences were collapsed to produce a dataset of unique, high-quality antibody sequences.

### Clustering of Antibody Repertoires

We perform agglomerative hierarchical clustering with complete linkage on both VDJ genes and tissue-specific donor pools. First, we perform a filter that removes VDJ genes with low counts of low variation across all samples. Then we calculate pairwise distances between genes and tissue-specific donor pools using Pearson correlation. We standardize the values in the heat map to display in the range −3 to +3. Dendrograms and heatmaps were created with Matlab R2010b.

### Analysis of Differential Expression of V(D)J Recombinants

We use the edgeR software [26] to calculate differential expression between tissues. EdgeR uses the negative binomial as the appropriate distribution for count data. We obtain dispersion estimates and test differential expression using the generalized linear model (GLM) likelihood ratio test. The columns in the table show the fold change between tissues and the p-value and Benjamini and Hochberg false discovery rate.

## Results

### Antibody variable gene use in peripheral blood, bone marrow or tissue repertoires

We obtained RNA from several pooled tissue samples: peripheral blood leukocytes, bone marrow, small intestine, lung, stomach, lymph node, tonsil, spleen and thymus. The number of donors and the age distribution for each tissue RNA pool are shown in Table 1. The antibody genes were amplified using RT-PCR, sequence adapters required for 454 sequencing and indexing barcodes were added during a second round of PCR, and the resulting amplicons were subjected to high-throughput DNA sequencing. Following initial sequence analysis using the IMGT High/V-QUEST server, we performed additional antibody-specific quality filtering. Antibody sequences were only retained for analysis if they were of the appropriate length (>300 bp); contained IMGT-assigned V, D and J genes; and an in-frame junction without ambiguous nucleotides. In addition, to reduce the repertoire-skewing effect of high-copy plasma cells, we removed redundant sequences. High copy number sequences resulting from plasma cells may still be over-represented, however. Due to sequencing and/or PCR errors, multiple reads of antibody sequences derived from a single plasma cell may not be identical and thus would not be removed during the filtering process. After filtering, we obtained a total of 1,412,943 unique antibody sequences. Read statistics for each tissue sample are given in Table 3.

**Table 3:** Read data from high-throughput sequence analysis of tissue RNA.

Sample	Number of reads	High quality reads	% High quality
Peripheral blood leukocytes	149,896	137,986	92.05
Bone marrow	171,111	159.515	93.22
Small intestine	118,044	137,986	93.45
Lung	198,660	183,264	92.25
Stomach	145,163	133,201	91.76
Lymph node	165,091	157,334	95.30
Tonsil	197,846	186,962	94.50
Spleen	185,247	175,689	94.84
Thymus	179,969	168.679	93.73
**Total**	1,511,027	1,412,943	93.51

Reads are wells that passed all standard 454 sequence analysis filters. Filtered reads are reads that passed additional antibody-related filters, including appropriate read length (>300 bases), identification of V, D, and J segments by IMGT analysis, and presence of an in-frame junctional rearrangement.

**Figure 1:**
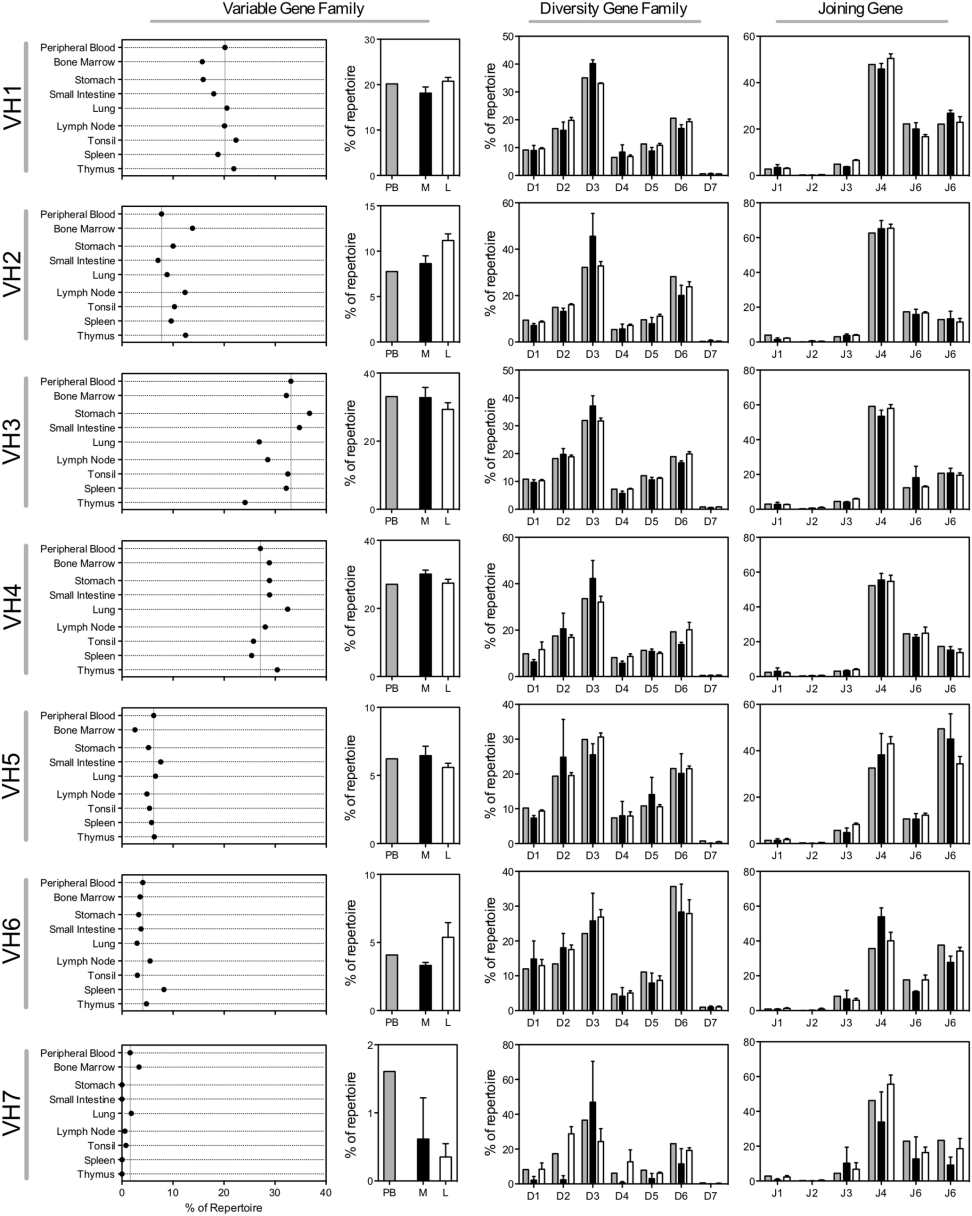
Germline gene family use. Starting from the left, the first column of panels shows the variable gene family use in peripheral blood, bone marrow, mucosal tissues (lung, small intestine, stomach) and lymphoid tissues (lymph node, tonsil, spleen and thymus). For easier comparison, the dashed vertical line in each panel represents the peripheral blood frequency. The second column of panels shows the variable gene use of peripheral blood and the combined variable gene family use of mucosal or lymphoid tissues. Bars indicate mean ± SEM for each group of tissue samples. The third column of panels shows the diversity gene family use in peripheral blood (grey bars), mucosal tissues (black bars) and lymphoid tissues (white bars). Bars indicate mean ± SEM for each group of tissue samples. The final column of panels shows the joining gene use. Colors are the same as the diversity gene family frequency panels. Bars indicate mean ± SEM for each group of tissue samples.

We first determined the frequency at which each variable gene family was found in each tissue and discovered substantial differences in the gene family use in tissues compared to peripheral blood (Figure 1). There are too many differences to detail individually, so we focus here on three of the most immediately apparent trends. First, variable gene family 2 (V_H_2) use was increased in every tissue except for small intestine, when compared to the peripheral blood repertoire. V_H_2 was found in 7.7% of peripheral blood sequences, and the largest increases were found in bone marrow (13.8%), thymus (12.5%) and lymph node (12.3%). Second, while V_H_3 was the most common antibody variable gene family in most of the samples, including peripheral blood as has been shown previously [13], [16], [17], the lung and thymus samples used the V_H_4 family more frequently than V_H_3. This finding was somewhat surprising, since V_H_3 has been shown to be the most frequently used germline gene throughout early B cell development and across peripheral blood B cell subsets [13], [16]. The substantial increase in use of V_H_4 in select tissue samples suggests strong selection of V_H_4 antibodies in these tissues. Finally, we found interesting contrasts when grouping tissue antibody repertoires into mucosal (stomach, lung, small intestine) and lymphoid (lymph node, spleen, tonsil, thymus) groups. All four lymphoid tissue samples showed reduced V_H_3 family use compared to peripheral blood, while two of the three mucosal tissue samples (stomach and small intestine) showed increased use of V_H_3 family genes. Analysis of the diversity (D) gene family and joining (J) gene use within each variable gene family produced similar usage patterns for both the mucosal and lymphoid groups.

### The V(D)J recombinant repertoire of mucosal tissues differed from that of peripheral blood or lymphoid tissues

**Figure 2:**
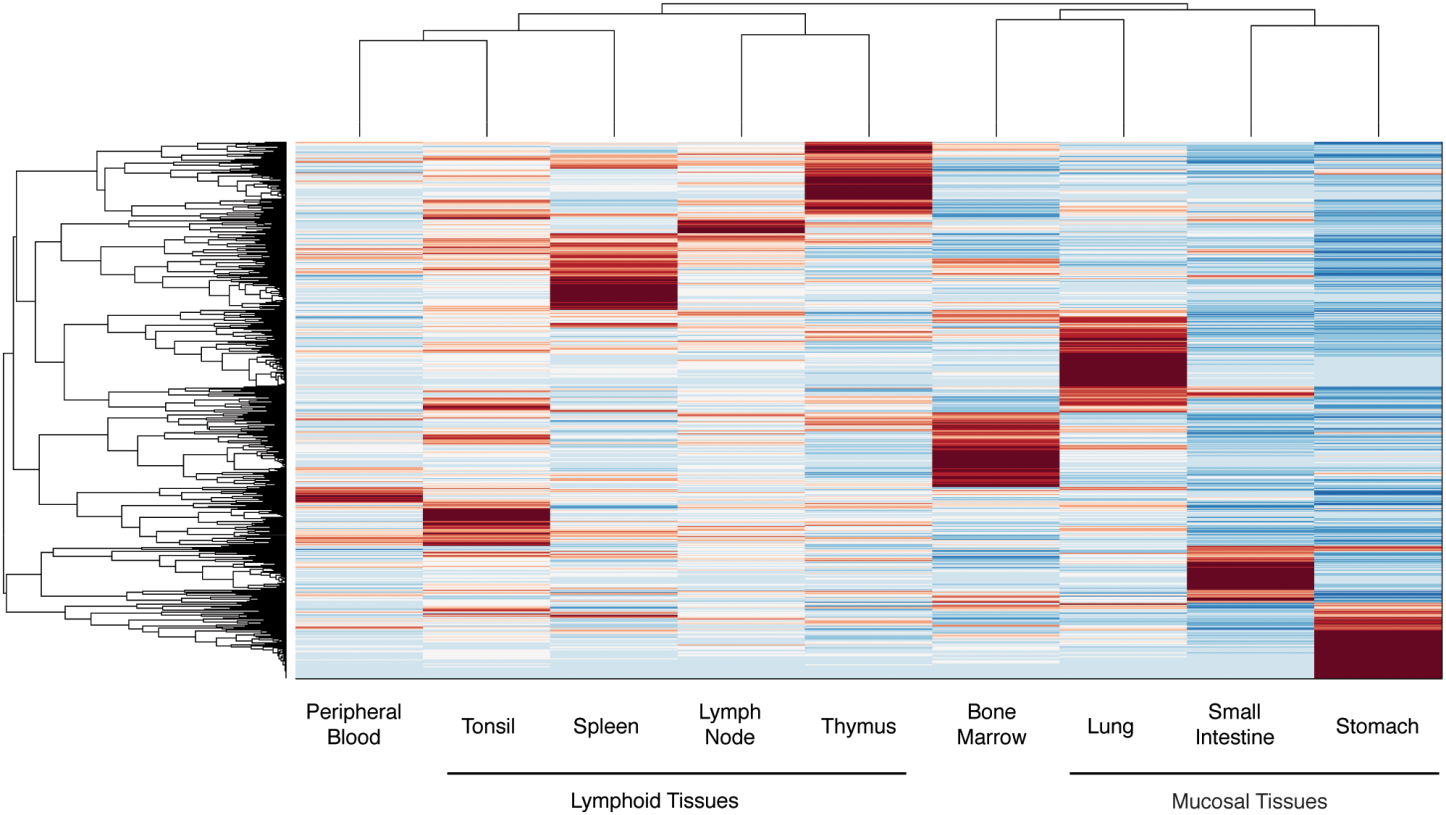
Clustergram of antibody gene repertoires. The frequency of each V_H_(D)J_H_ recombination was determined for each of nine tissues, and a clustergram was created. V_H_(D)J_H_ recombinants were clustered by relative frequency in each tissue-specific repertoire, and the resulting phylogenetic tree is shown on the left. Tissue-specific repertoires were clustered by the overall V_H_(D)J_H_ usage of each repertoire, and the resulting clustering diagram is shown at the top. The frequency variation for each V_H_(D)J_H_ recombination across all tissue-specific repertoires was determined, and standardized to a range of −3 to 3. A complete list of the frequency variation of all V_H_(D)J_H_ recombinants for each tissue-specific repertoire, along with statistical significance and false discovery rate (FDR) calculations, is available in File S1 (see eight files, each named Suppl Info_PB_vs_*tissue*).

We next performed a hierarchical clustering analysis on the V(D)J recombinant repertoire for each tissue sample (Figure 2). Interestingly, lymphoid tissues (tonsil, spleen, lymph node and thymus) clustered with each other and with peripheral blood. Mucosal tissues (lung, small intestine, stomach) also clustered together, along with bone marrow. This analysis indicates that the architecture of V(D)J recombinant repertoires of mucosal tissues differs substantially from both peripheral blood and lymphoid tissue repertoires. It is also interesting that the mucosal tissue repertoires, which have been shown to be composed primarily of B cells encoding antigen-specific antibodies [27], cluster with the bone marrow samples, in which the overwhelming majority of transcription is performed by long-lived plasma cells which are thought to produce much of the circulating antibody proteins in serum. These data suggests that antibody repertoires encoded by tissue-specific B cells may better represent the repertoire of circulating serum antibody proteins than are the antibody repertoires encoded by B cells circulating in the peripheral blood.

**Figure 3:**
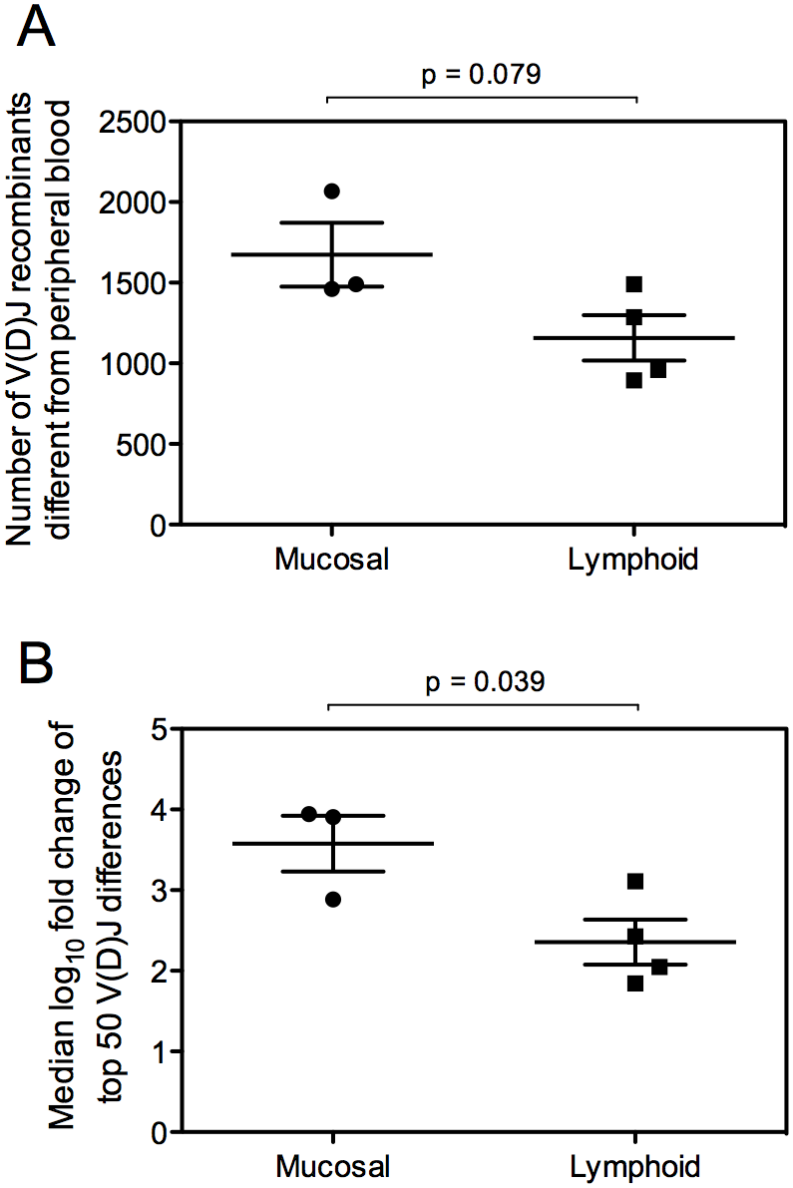
Comparison of V_H_(D)J_H_ use in lymphoid or mucosal tissues to that in peripheral blood. (A) The frequency of each V_H_(D)J_H_ recombination was calculated for each tissue and compared to peripheral blood. The number of V_H_(D)J_H_ recombinants for which the frequency differed significantly from peripheral blood was calculated for each tissue (statistical false discovery rate (FDR) calculations are available in File S1 [see eight files, each named Suppl Info_PB_vs_*tissue]*). The number of statistically different V_H_(D)J_H_ combinations is shown for each mucosal (lung, small intestine, stomach) and lymphoid (lymph node, tonsil, spleen and thymus) tissue. (B) The frequency of each V_H_(D)J_H_ recombination was determined for each tissue and compared to peripheral blood. The fold change of the 50 most different V_H_(D)J_H_ recombinations is shown in log_10_ scale for each tissue.

To more closely investigate these repertoire differences, we determined the frequency of each V(D)J combination and, for each tissue repertoire sample, calculated the number of V(D)J combinations for which the frequency differed statistically from the peripheral blood sample (Figure 3A and Table S1). We found a trend toward more differences between mucosal tissue samples and peripheral blood than were present between lymphoid tissue samples and blood (p = 0.079). We also analyzed the magnitude of the top 50 differences from peripheral blood for each of the mucosal and lymphoid samples, and found that differences in V(D)J frequency between mucosal tissue samples and peripheral blood were significantly larger than differences between lymphoid tissue samples and peripheral blood (Figure 3B; p = 0.039). Thus, the genetic composition of mucosal tissue antibody repertoires differs more substantially from peripheral blood than it does from lymphoid tissue repertoires.

### Mutation frequency analysis of peripheral blood, bone marrow or tissue repertoires

**Figure 4:**
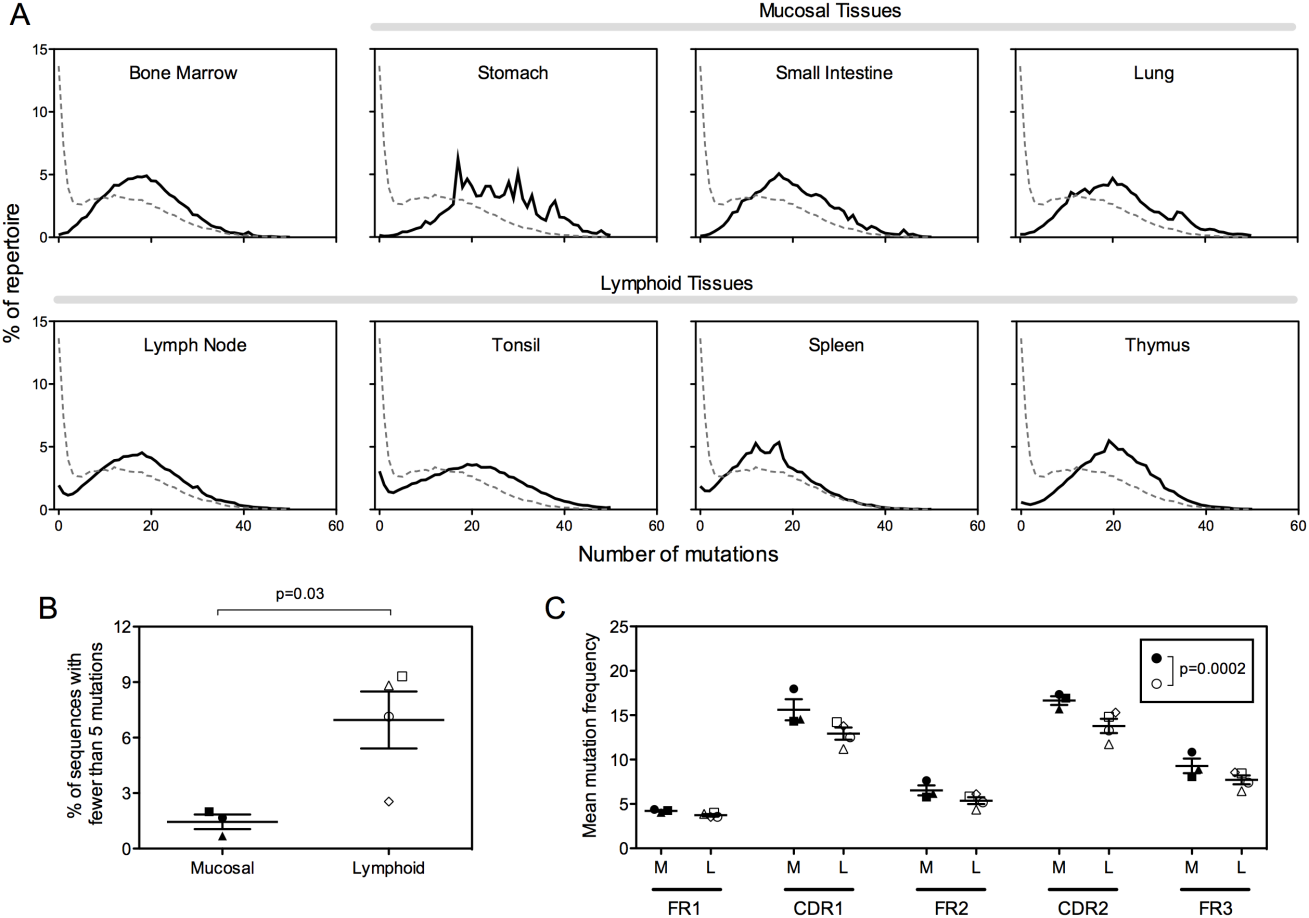
Mutation frequency for peripheral blood, bone marrow and mucosal and lymphoid tissues. (A) Mutation histograms are shown for each sample. Each of the three mucosal tissue samples (small intestine, stomach and lung) shows a complete loss of un-mutated sequences, which constitute a large portion of the peripheral blood repertoire. Repertoires for each of the lymphoid tissues (lymph node, tonsil, spleen and thymus) contained antibody genes with few or no mutations, but at a lower frequency than peripheral blood. For ease of comparison, the mutation distribution for peripheral blood is shown as a dashed line in each tissue plot. (B) The frequency of sequences with fewer than 5 mutations was determined for each mucosal and lymphoid tissue sample. For mucosal samples, lung, small intestine or stomach samples are plotted as filled circles, squares or triangles, respectively. For lymphoid samples, lymph node, spleen, thymus or tonsil are plotted as open circles, squares, triangles and diamonds, respectively. (C) The mean mutation frequency is shown for each genetic region of the variable gene: Framework Regions 1, 2 and 3 (FR1, FR2, FR3) and Complementarity Determining Regions 1 and 2 (CDR1, CDR2). Bars indicate mean ± SEM for each group of tissue samples. Sample glyphs are as in (B).

Sequences from each tissue subset were grouped by mutation frequency. Then, the relative abundance of each mutation frequency group was calculated, and a mutation histogram was created for each tissue sample (Figure 4A). The peripheral blood sample contained a large number of sequences with few or no mutations, as has been shown previously [13], [16]. The bone marrow sample contained very few sequences with few or no mutations, which is somewhat surprising, since bone marrow contains many progenitor and precursor B cells, which are presumably un-mutated. The low frequency of un-mutated sequences is likely due to the abundance of plasma cell transcripts among the RNA used as template for antibody gene amplification. Since bone marrow resident long-lived plasma cells transcribe the antibody gene at a much higher rate than immature B cells, it is likely that oversampling of RNA transcripts derived from plasma cells skewed the bone marrow sequence repertoire toward highly mutated sequences. In this sense, the bone marrow antibody sequences reported here are more likely to reflect the composition of plasma antibody repertoires, which are composed mainly of antibody produced by long-lived plasma cells, than a representation of the early developing B cell population.

All three mucosal tissue samples (small intestine, stomach and lung) showed a dramatically lower frequency of sequences with few or no mutations, suggesting that naïve B cells are less frequent in tissue. Interestingly, the lymphoid tissues (lymph node, tonsil, spleen and thymus) contained a higher frequency of antibody genes with few or no mutations (mean = 7.0%) than mucosal tissues (1.4%; p = 0.03), but a much lower frequency than circulating B cells in the peripheral blood (30.6%; Figure 4B). Here again, we observed a similarity between the bone marrow and mucosal tissue repertoires. As with V(D)J gene use, the mutation frequency and abundance of un-mutated sequences in the bone marrow repertoire was similar to each of the mucosal tissue repertoires and differed substantially from repertoires in lymphoid tissue and peripheral blood.

A more detailed breakdown of mutation frequency by antibody gene region (Figure 4C) shows a reduction in mutation frequency in lymphoid tissue repertoires across all framework regions (FRs) and complementarity determining regions (CDRs) when compared to mucosal tissue repertoires (p = 0.0002).

### Mucosal tissue repertoires encode longer HCDR3s than lymphoid tissue repertoires

**Figure 5:**
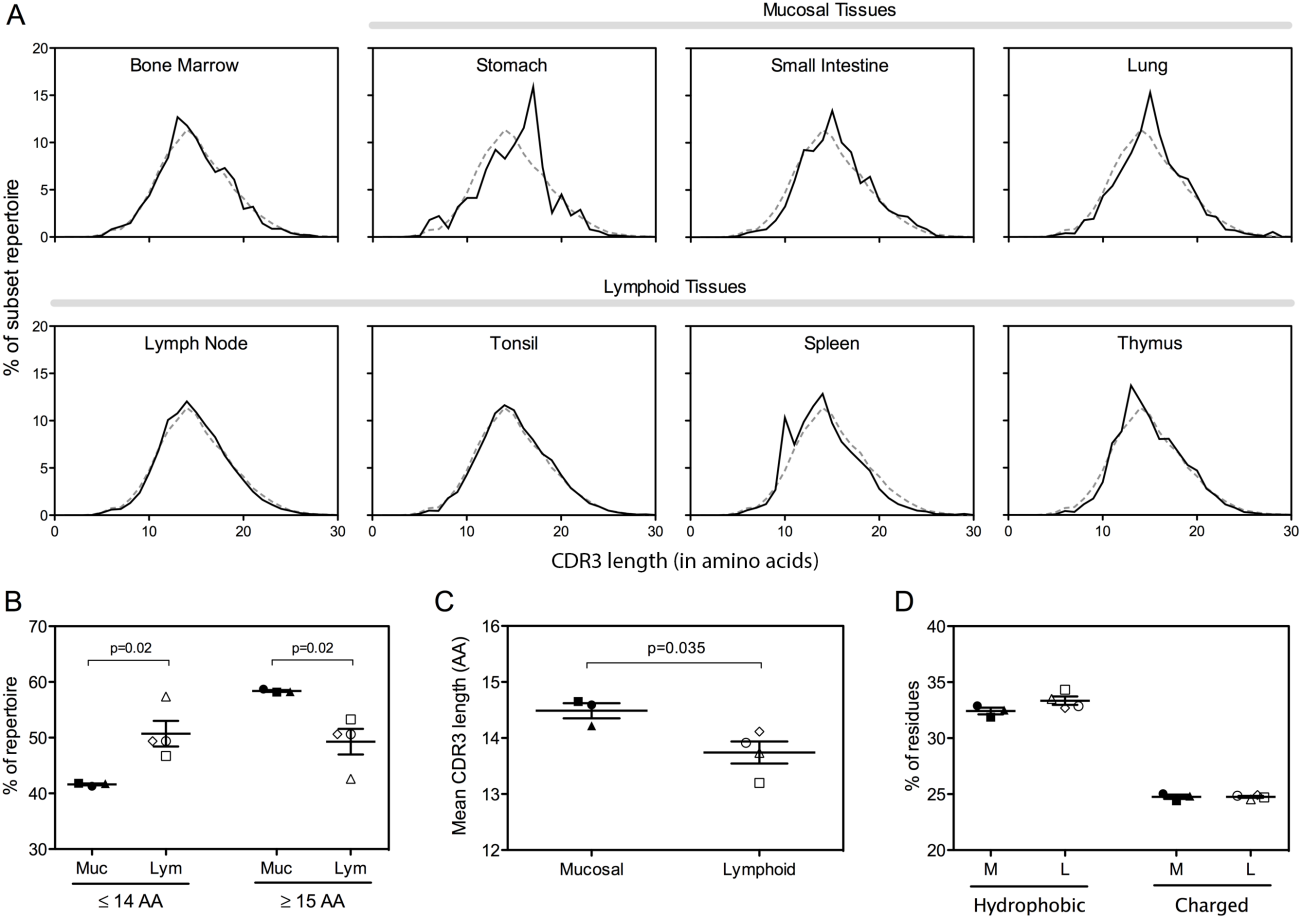
Mucosal tissue antibody gene repertoires encode longer CDR3s and are more mutated than lymphoid tissue repertoires. (A) Heavy chain CDR3 length histograms for each tissue sample. For ease of comparison, the CDR3 length distribution for peripheral blood is shown as a dashed line in each tissue plot. (B) Frequency of sequences containing short (14AA or shorter) or long (15AA or longer) CDR3s. Bars indicate mean ± SEM for each group of samples. (C) The mean CDR3 length was determined for each tissue-specific repertoire. Bars indicate mean ± SEM for each group of samples. For mucosal samples, lung, small intestine or stomach samples are plotted as filled circles, squares and triangles, respectively. For lymphoid samples, lymph node, spleen, thymus and tonsil or plotted as open circles, squares, triangles and diamonds, respectively. (D) Frequency of hydrophobic and charged CDR3 residues. Bars indicate mean ± SEM for each group of samples. Samples glyphs are as in (C).

Sequences from each tissue repertoire were grouped by HCDR3 length and the frequency of each HCDR3 length group was determined (Figure 5A). To facilitate comparisons, the HCDR3 length histogram for the peripheral blood repertoire is displayed alongside each tissue HCDR3 histogram. Tissue repertoires then were divided into two groups based on HCDR3 length: short HCDR3s (≤14 amino acids; shorter than the mean HCDR3 length in the peripheral blood repertoire) and long HCDR3s (≥15 amino acids; longer than the mean HCDR3 length in the peripheral blood repertoire) (Figure 5B). The repertoires of lymphoid tissues were approximately evenly split between short (50.7%) and long (49.3%) HCDR3 lengths. In contrast, repertoires from mucosal tissues contained a significantly higher frequency of long HCDR3s (58.4%) and thus a significantly lower frequency of short HCDR3s (41.6%) than lymphoid repertoires (p = 0.02). Further, the overall mean HCDR3 length of the mucosal tissue repertoires was significantly longer than the overall mean HCDR3 length of lymphoid tissue repertoires (Figure 5C; p = 0.035). This finding was surprising since mucosal repertoires contain a higher fraction of highly mutated sequences than lymphoid repertoires (Figure 4), but the highly mutated memory B cell subsets have been shown to encode shorter HCDR3s than the un-mutated naïve subset [16], [28]. Since long HCDR3s tend to have a lower frequency of hydrophobic and charged residues [29]–[33], we determined the frequency of both hydrophobic and charged HCDR3 residues in mucosal and lymphoid repertoires (Figure 5D). While there was a trend toward reduced frequency of hydrophobic HCDR3 residues in the mucosal tissue repertoires compared to lymphoid repertoires (p = 0.13), there was no difference in the frequency of charged HCDR3 residues in mucosal repertoires (23.4%) compared to lymphoid repertoires (23.2%; p = 0.61).

### Somatic hypermutation-associated insertions and deletions in peripheral blood, mucosal tissue and lymphoid tissue repertoires

**Figure 6:**
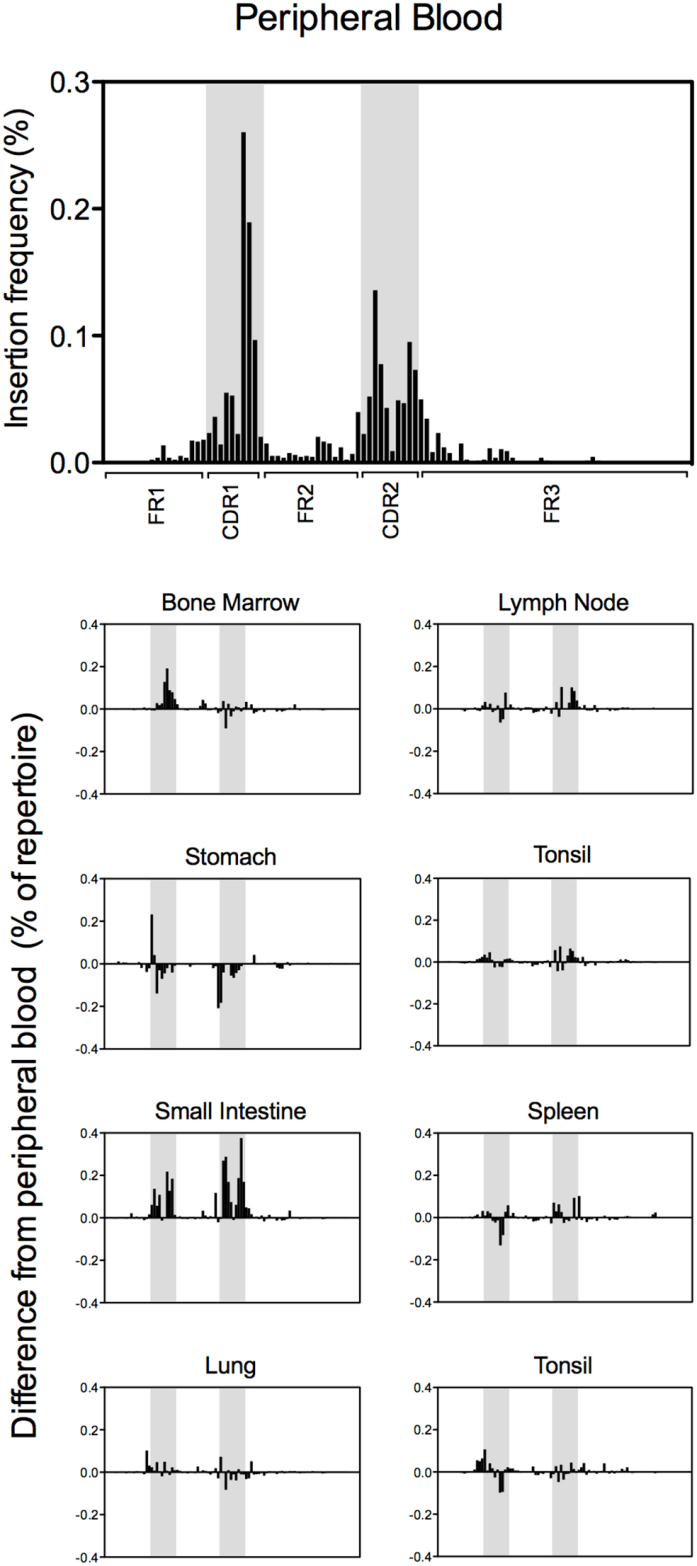
Frequency and position of DNA fragments encoding non-frameshift insertions. (A) The presence and frequency of non-frameshift insertions is shown for the peripheral blood repertoire. The frequency is plotted as the percent of sequences in the repertoire displaying deletions for each codon position in the variable gene. The location of CDR1 and CDR2 are highlighted in grey. (B) The difference in insertion frequency compared to peripheral blood is shown for each tissue. As in (A), CDR1 and CDR2 are highlighted in grey.

**Figure 7:**
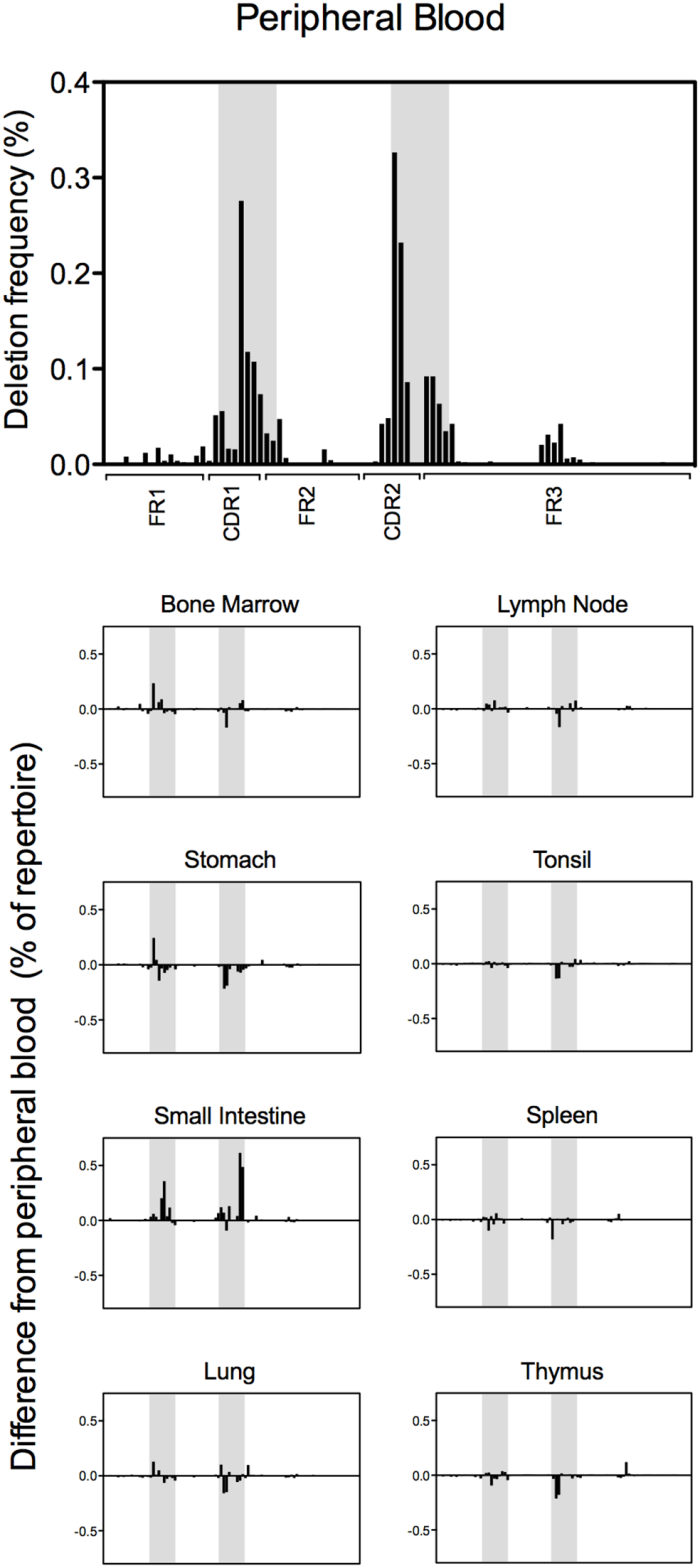
Frequency and position of DNA fragments encoding non-frameshift deletions. (A) The presence and frequency of non-frameshift deletions is shown for the peripheral blood subset. The frequency is plotted as the percent of sequences in the repertoire displaying deletions for each codon position in the variable gene. The location of CDR1 and CDR2 are highlighted in grey. (B) The difference in deletion frequency when compared to that in peripheral blood is shown for each tissue. As in (A), CDR1 and CDR2 are highlighted in grey.

Short nucleotide insertions or deletions have been shown to be associated with the somatic hypermutation process and antibodies encoding these somatic hypermutation-associated insertions and deletions (SHA indels) have been shown to be critical to the immune response against pathogens that initiate infection at mucosal surfaces [34]–[40]. The heavy chain sequences of antibodies from all tissue repertoires were analyzed for the presence of codon-length nucleotide insertions or deletions in the antibody variable gene region. Sequences from the peripheral blood repertoire containing SHA indels were analyzed further to determine the position of each SHA indel, and the frequency of insertions (Figure 6) or deletions (Figure 7) at each codon position of the variable gene was calculated. For each mucosal or lymphoid tissue repertoire, the difference in SHA indel frequency, compared to peripheral blood, was calculated at each codon position. Insertions and deletions were both located predominantly in CDRs as opposed to framework regions, and were distributed roughly equally between heavy chain CDR1 and heavy chain CDR2 loops.

A large increase in frequency of both insertions and deletions in the small intestine antibody repertoire was immediately apparent. This observation was even more surprising since the frequency of SHA indels has been shown to correlate with the frequency of somatic hypermutation events [41], but a corresponding increase in somatic mutations was not seen in the small intestine antibody repertoire (Figure 4A). Thus, the large increase in SHA indel frequency is likely due to specific, antigen-driven enrichment in the small intestine repertoire, not as an indirect result of a more general increase in somatic hypermutation events.

## Discussion

The substantial differences discovered in mucosal tissue repertoires compared to peripheral blood and lymphoid tissue repertoires suggest that mucosal tissues contain B cells that express a unique, specialized repertoire of antibody genes, possibly to mount a more effective response to infections that are initiated at mucosal surfaces. The genetic composition of antibody repertoires encoded by circulating naive and memory B cells have been shown to differ, but the repertoires between naive and memory subsets were observed previously to be much more similar than expected. Further, memory repertoires have been shown to be very similar across individuals. While it is unclear how the consistency of the memory repertoire is maintained over the course of multiple pathogenic encounters that each induce biased antibody responses, we hypothesized that some fraction of the pathogen-induced memory population may be segregated into the mucosal tissue that represents the initial site of pathogen contact. To test this hypothesis, we performed a detailed genetic analysis of the expressed antibody repertoires of peripheral blood, bone marrow, and various mucosal and lymphoid tissues. Although we cannot define the root cause of the repertoire differences – likely due a combination of factors, including altered B cell subset composition, exposure to unique pathogenic challenges, and distinct antibody maturation mechanisms – our findings suggest that mucosal tissues harbor unique tissue-specific repertoires.

We found several genetic characteristics, including germline gene use, mutation frequency and HCDR3 length, for which the mucosal tissue repertoires substantially differed from lymphoid tissue and peripheral blood repertoires. When performing hierarchical clustering on the V(D)J use in each sample, we found that all mucosal tissue repertoires clustered together, separate from peripheral blood and lymphoid tissue repertoires. Interestingly, the two mucosal tissues that would be considered most similar, the stomach and small intestine samples, were identified as the mucosal samples containing the most closely related antibody repertoire. Analysis of mutation frequency in each sample revealed the nearly complete absence of un-mutated sequences in the mucosal tissue repertoires, in stark contrast to the relatively high frequency of sequences with few or no mutations found in lymphoid tissue and peripheral blood repertoires. Finally, HCDR3 analysis revealed striking differences between mucosal and lymphoid tissues. Lymphoid tissues were most similar to the peripheral blood repertoire, with about half of each lymphoid tissue repertoire encoding HCDR3s that were as long or longer than the mean HCDR3 length in the peripheral blood repertoire (15 amino acids). In contrast, nearly 60% of sequences in each of the mucosal tissue repertoires were 15 amino acids in length or longer, indicating strong selection pressure for long HCDR3s in the mucosal repertoires. These data suggest that mucosal antibody repertoires may be tuned specifically to respond effectively to a subset of pathogens likely to be encountered at the mucosal interface.

In some sense, comparing the genetic composition of these repertoires is akin to comparing apples and oranges: peripheral blood primarily consists of mature naïve B cells, while the tissue samples likely contain a far higher fraction of memory B cells and antibody secreting cells. However, the observed differences cannot entirely be explained by the varying B cell subset proportions. Although variable gene use differed substantially between tissue and peripheral blood samples, diversity and joining gene use was statistically indistinguishable, indicating the presence of selective pressure on variable gene use that extends beyond B cell subset composition. In addition, the two tissues that would be expected to contain the most similar proportion of B lineage cells, stomach and intestine, show striking differences that defy their presumably similar repertoire composition. If the differences between the tissue and peripheral blood repertoires were due primarily to the altered B cell subset contribution, it would be expected that known differences between the primary components of the peripheral blood repertoire (mature naïve B cells) and the tissue repertoires (memory B cells and plasmablasts) would be similar to those seen between the entire repertoires. For example, it has been repeatedly shown that memory B cells and plasmablasts have shorter HCDR3s than mature naïve B cells [16], [27], [28]. In the tissue repertoires, however, we saw the opposite: mucosal tissue repertoires, which would be expected to have shorter HCDR3s than peripheral blood due to increased memory B cell and plasmablast frequency, encoded significantly longer HCDR3s that the peripheral blood and lymphoid tissue repertoires. While it is clear that some of the observed differences are due to the dissimilar B cell subset makeup of tissue and peripheral blood samples, it is equally clear that the subset differences are insufficient to explain the entire difference.

While the differences between the mucosal tissue repertoires and peripheral blood were unexpected, more surprising were the observed similarities between mucosal tissue repertoires and the expressed repertoire of the bone marrow. At the cellular level, much of the bone marrow B cell population consists of progenitor and precursor B cells in the early stages of development. In contrast, long-lived plasma cells, which produce orders of magnitude more antibody transcript per cell than developing B cells, contribute a disproportionately large portion of the bone marrow antibody RNA pool. Because long-lived plasma cells are thought to produce the majority of soluble antibody proteins in serum and other body fluids, the genetic composition of the bone marrow antibody mRNA pool would be expected to provide a reasonable estimation of the soluble plasma antibody repertoire. Our data, combined with recent proteomics evidence showing that the serum antibody repertoire differs from the antibody repertoire encoded by circulating B cells [42], suggest that genetic and functional antibody studies must expand their focus beyond the peripheral blood antibody repertoire. Although further work must be done to more completely define the antigen-specific nature of mucosal antibody repertoires, the work presented here provides a valuable step towards a more complete understanding of the entire humoral immune response.

## Supporting Information

Table S1 Number and fold change of V(D)J combinations for each tissue repertoire sample for which the frequency differed statistically from the peripheral blood sample.
(PDF)

File S1 Suppl Info_PB_vs_Bone_marrow.csv.txt. Suppl Info_PB_vs_Lung.csv.txt. Suppl Info_PB_vs_Lymph_node.csv.txt. Suppl Info_PB_vs_Small_intestine.csv.txt. Suppl Info_PB_vs_Spleen.csv.txt. Suppl Info_PB_vs_Stomach.csv.txt. Suppl Info_PB_vs_Thymus.csv.txt. Suppl Info_PB_vs_Tonsil.csv.txt.
(ZIP)
