# Correction: Tissue-Specific Expressed Antibody Variable Gene Repertoires

**DOI:** 10.1371/journal.pone.0228412

**Published:** 2020-01-24

**Authors:** Bryan S. Briney, Jordan R. Willis, Jessica A. Finn, Brett A. McKinney, James E. Crowe

Fig 1 is incorrect. The authors have provided a corrected version here.

**Fig 1:**
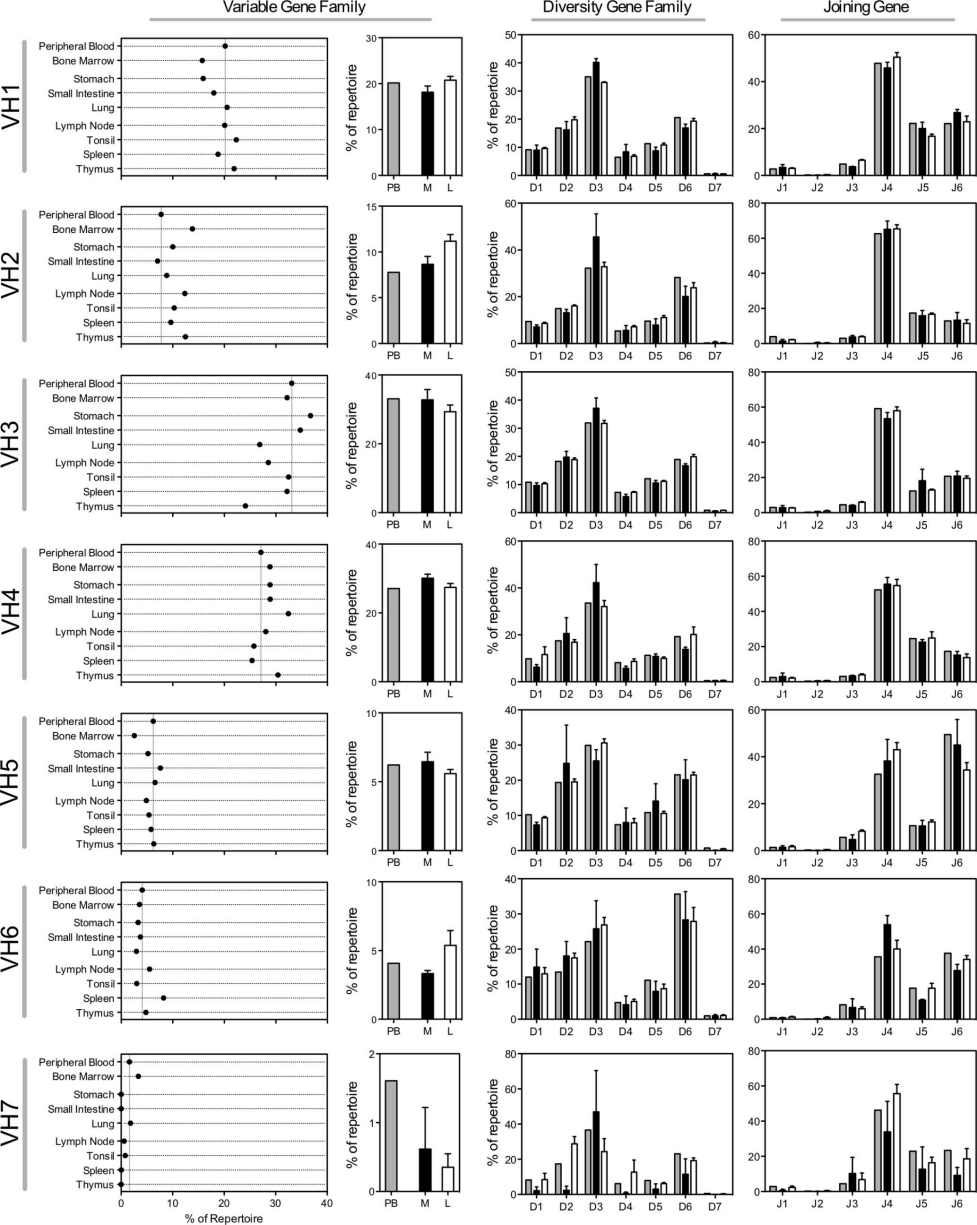
Germline gene family use. Starting from the left, the first column of panels shows the variable gene family use in peripheral blood, bone marrow, mucosal tissues (lung, small intestine, stomach) and lymphoid tissues (lymph node, tonsil, spleen and thymus). For easier comparison, the dashed vertical line in each panel represents the peripheral blood frequency. The second column of panels shows the variable gene use of peripheral blood and the combined variable gene family use of mucosal or lymphoid tissues. Bars indicate mean ± SEM for each group of tissue samples. The third column of panels shows the diversity gene family use in peripheral blood (grey bars), mucosal tissues (black bars) and lymphoid tissues (white bars). Bars indicate mean ± SEM for each group of tissue samples. The final column of panels shows the joining gene use. Colors are the same as the diversity gene family frequency panels. Bars indicate mean ± SEM for each group of tissue samples.
