# Supplementary material for: Tissue-Specific Expressed Antibody Variable Gene Repertoires
Source: PLoS One. 2014 Jun 23;9(6):e100839. doi: 10.1371/journal.pone.0100839 (PMC4067404; doi:10.1371/journal.pone.0100839)
Supplement: Table S1 — Number and fold change of V(D)J combinations for each tissue repertoire sample for which the frequency differed statistically from the peripheral blood sample. (PDF) [file pone.0100839.s001.pdf]

Table S1. Number and fold change of V(D)J combinations for each tissue repertoire sample for which the frequency differed statistically from the peripheral blood sample.

	**Region**	**Specific Tissue**	**Number of V(D)J recombinants different from peripheral blood**
**Figure 3A**	Mucosal	Lung	1,461
		Small Intestine	1,491
		Stomach	2,068
	Lymphoid	Thymus	1,491
		Lymph Node	895
		Spleen	1,287
		Tonsil	960
	**Region**	**Specific Tissue**	**Median $\log_{10}$ fold change of top 50 V(D)J differences**
**Figure 3B**	Mucosal	Lung	767
		Small Intestine	8,035
		Stomach	8,790
	Lymphoid	Thymus	267
		Lymph Node	111
		Spleen	1,291
		Tonsil	69
